# 6,6′-Dihydr­oxy-2,2′-[(butane-1,4-diyldi­oxy)bis­(nitrilo­methyl­idyne)]diphenol

**DOI:** 10.1107/S1600536808028468

**Published:** 2008-09-13

**Authors:** Wen-Kui Dong, Xue-Ni He, Yin-Xia Sun, Li Xu, Yong-Hong Guan

**Affiliations:** a School of Chemical and Biological Engineering, Lanzhou Jiaotong University, Lanzhou 730070, People’s Republic of China

## Abstract

The mol­ecule of the centrosymmetric title compound, C_18_H_20_N_2_O_6_, assumes an *E* configuration with respect to the azomethine C=N bond. The imino group is coplanar with the aromatic ring. Intra­molecular O—H⋯O and O—H⋯N bonds are found between the hydroxyl groups and adjacent O (or N) atoms. In the crystal structure, inter­molecular O—H⋯O bonds link each mol­ecule to two others, forming a layered network.

## Related literature

For background information, see: Sharma (2002 ▶). For related structures, see: Fan *et al.* (2006 ▶); Wang *et al.* (2003 ▶); Akine *et al.* (2006 ▶). Dong *et al.* (2007 ▶, 2008*a* ▶,*b* ▶); Wang *et al.* (2007 ▶).
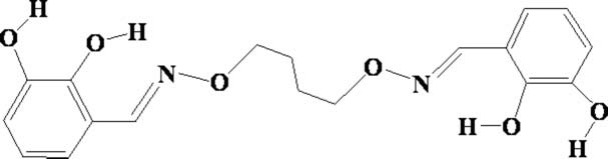


## Experimental

### 

#### Crystal data

C_18_H_20_N_2_O_6_
*M* *_r_* = 360.36
Mmonoclinic, 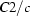

*a* = 27.484 (3) Å
*b* = 4.7106 (7) Å
*c* = 14.0081 (19) Å
β = 104.306 (2)°
*V* = 1757.4 (4) Å^3^
*Z* = 4
Mo *K*α radiation
μ = 0.10 mm^−1^
*T* = 298 (2) K
0.55 × 0.53 × 0.48 mm

#### Data collection

Siemens Smart 1000 CCD area-detector diffractometer
Absorption correction: multi-scan (*SADABS*; Sheldrick, 1996 ▶) *T*_min_ = 0.945, *T*_max_ = 0.952
4086 measured reflections
1555 independent reflections
1112 reflections with *I* > 2σ(*I*)
*R*_int_ = 0.026

#### Refinement

*R*[*F* ^2^ > 2σ(*F* ^2^)] = 0.035
*wR*(*F* ^2^) = 0.106
*S* = 1.10
1555 reflections
118 parameters
H-atom parameters constrained
Δρ_max_ = 0.16 e Å^−3^
Δρ_min_ = −0.15 e Å^−3^

### 

Data collection: *SMART* (Siemens, 1996 ▶); cell refinement:  *SAINT* (Siemens, 1996 ▶); data reduction: *SAINT*; program(s) used to solve structure: *SHELXS97* (Sheldrick, 2008 ▶); program(s) used to refine structure: *SHELXL97* (Sheldrick, 2008 ▶); molecular graphics: *SHELXTL* (Sheldrick, 2008 ▶); software used to prepare material for publication: *SHELXTL*.

## Supplementary Material

Crystal structure: contains datablocks global, I. DOI: 10.1107/S1600536808028468/fl2218sup1.cif

Structure factors: contains datablocks I. DOI: 10.1107/S1600536808028468/fl2218Isup2.hkl

Additional supplementary materials:  crystallographic information; 3D view; checkCIF report

## Figures and Tables

**Table 1:** Hydrogen-bond geometry (Å, °)

*D*—H⋯*A*	*D*—H	H⋯*A*	*D*⋯*A*	*D*—H⋯*A*
O2—H2⋯N1	0.82	1.94	2.648 (2)	145
O3—H3⋯O2	0.82	2.26	2.706 (2)	115
O3—H3⋯O1^i^	0.82	2.26	2.930 (2)	139

Symmetry code: (i) 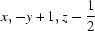.

**Fig. 1.**
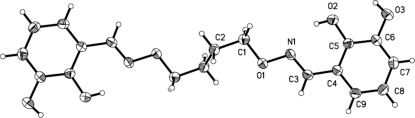
The molecular structure of the title compound with atom numbering scheme [Symmetry codes: -x,-y + 2,-z + 1]. Displacement ellipsoids for non-hydrogen atoms are drawn at the 30% probability level.

**Fig. 2.**
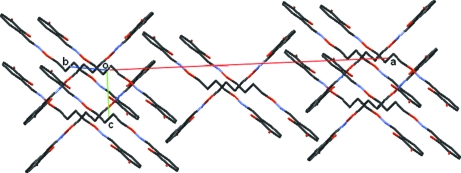
Three-dimensional packing diagram of the title compound along c axis showing the E configuration.

**Fig. 3.**
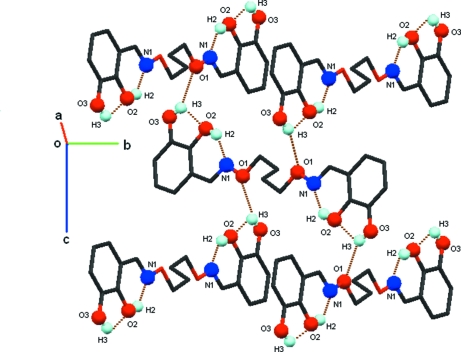
Part of the supramolecular structure of the title compound along b axis. Intra- and intermolecular hydrogen bonds are shown as dashed lines.

## supplementary crystallographic information

### Comment

The design of supramolecular structures involves molecules for which intermolecular hydrogen bonds act as driving, directional and cohesive forces (Sharma, 2002). Although many stable and well documented structures have been reported (Fan *et al.*, 2006; Wang *et al.*, 2003), the supramolecular structures of salen-type bisoxime compounds have rarely been determined. The first reported supramolecular salen-type bisoxime compound, 6,6'-dihydroxy-2,2'-[ethylenedioxybis(nitrilomethylidyne)]diphenol has an intermolecular hydrogen bond network involving four hydroxyl groups and cocrystallized water molecules (Akine *et al.*, 2006). In this article, we report the synthesis and structure of the title compound (I) (Fig. 1).

(I) lies across a crystallographic inversion centre to give 1/2 molecule per asymmetric unit. It assumes an E configuration with respect to the azomethine C═N bond. The imino group is coplanar with the aromatic ring. The planar units are parallel to one another but extend in opposite directions from the tetramethylene bridge. Strong intramolecular O(3)—H(3)···O(2) and O(2)—H(2)···N(1) bonds are found between the hydroxyl groups and adjacent O (or N) atoms (Table 1). This is similar to what was observed in our previously reported salen-type bisoxime compounds (Wang *et al.*, 2007; Dong *et al.*, 2007; Dong *et al.*, 2008*a*).

In the crystal packing weak intermolecular O—H···O hydrogen bonds link each molecule to 4 others, forming an infinite three-dimensional supramolecular structure (Figs. 2 and 3) that differs from the structures of 6,6'-dihydroxy-2,2'-[ethylenedioxybis(nitrilomethylidyne)]diphenol (Akine *et al.*, 2006) and 6,6'-dihydroxy-2,2'-[(pentane-1,5-diyldioxy)bis(nitrilomethylidyne)]diphenol (Dong *et al.*, 2008*b*), in which the molecules exhibit one-dimensional chains formed through strong intermolecular π–π stacking interactions or weak intermolecular hydrogen bonds.

### Experimental

The title compound was synthesized according to an analogous method reported earlier (Dong *et al.*, 2007). To an ethanol solution (5 ml) of 2,3-dihydroxybenzaldehyde (276.6 mg, 2.0 mmol) was added an ethanol solution (5 ml) of 1,4-bis(aminooxy)butane (120.0 mg, 1.0 mmol). After the solution had been stirred at 328 K for 3 h, the reaction mixture was separated by filtration, washed successively with ethanol and ethanol/hexane (1:4), respectively. The product was dried under reduced pressure and purified with recrystallization from ethanol to yield 59.9 mg of pale-brown crystalline solid. Yield, 17.2%, m.p. 388–389 K. Anal. Calc. (%) for C_18_H_20_N_2_O_6_: C, 59.99; H, 5.59; N, 7.77. Found: C, 60.23; H, 5.45; N, 7.60.

Block-shaped crystals of (I) suitable for X-ray crystal analysis were grown from a mixture of tetrahydrofuran/ethanol (1:1) solution by slow evaporation at room temperature, which afforded pale-brown crystals.

### Refinement

Non-H atoms were refined anisotropically. H atoms were treated as riding atoms with distances C—H = 0.97 (CH_2_), 0.93 Å (CH), O—H = 0.82 Å and *U*_iso_(H) = 1.2 *U*_eq_(C) and 1.5 *U*_eq_(O).

### Crystal data

**Table:**

C_18_H_20_N_2_O_6_	*Z* = 4
*M**_r_* = 360.36	*F*(000) = 760
Mmonoclinic, *C*2/*c*	*D*_x_ = 1.362 Mg m^−^^3^
Hall symbol: -C 2yc	Mo *K*α radiation, λ = 0.71073 Å
*a* = 27.484 (3) Å	Cell parameters from 1695 reflections
*b* = 4.7106 (7) Å	θ = 2.4–27.8°
*c* = 14.0081 (19) Å	µ = 0.10 mm^−^^1^
α = 90°	*T* = 298 K
β = 104.306 (2)°	Block-shaped, pale-brown
γ = 90°	0.55 × 0.53 × 0.48 mm
*V* = 1757.4 (4) Å^3^	

### Data collection

**Table:**

Bruker Smart 1000 CCD area-detector diffractometer	1555 independent reflections
Radiation source: fine-focus sealed tube	1112 reflections with *I* > 2σ(*I*)
graphite	*R*_int_ = 0.026
φ and ω scans	θ_max_ = 25.0°, θ_min_ = 1.5°
Absorption correction: multi-scan (SADABS; Sheldrick, 1996)	*h* = −17→32
*T*_min_ = 0.945, *T*_max_ = 0.952	*k* = −5→5
4086 measured reflections	*l* = −16→16

### Refinement

**Table:**

Refinement on *F*^2^	Primary atom site location: structure-invariant direct methods
Least-squares matrix: full	Secondary atom site location: difference Fourier map
*R*[*F*^2^ > 2σ(*F*^2^)] = 0.036	Hydrogen site location: inferred from neighbouring sites
*wR*(*F*^2^) = 0.106	H-atom parameters constrained
*S* = 1.10	*w* = 1/[σ^2^(*F*_o_^2^) + (0.0457*P*)^2^ + 0.5441*P*] where *P* = (*F*_o_^2^ + 2*F*_c_^2^)/3
1555 reflections	(Δ/σ)_max_ < 0.001
118 parameters	Δρ_max_ = 0.16 e Å^−^^3^
0 restraints	Δρ_min_ = −0.16 e Å^−^^3^

### Special details

**Table:**

Geometry. All e.s.d.'s (except the e.s.d. in the dihedral angle between two l.s. planes) are estimated using the full covariance matrix. The cell e.s.d.'s are taken into account individually in the estimation of e.s.d.'s in distances, angles and torsion angles; correlations between e.s.d.'s in cell parameters are only used when they are defined by crystal symmetry. An approximate (isotropic) treatment of cell e.s.d.'s is used for estimating e.s.d.'s involving l.s. planes.
Refinement. Refinement of *F*^2^ against ALL reflections. The weighted *R*-factor *wR* and goodness of fit *S* are based on *F*^2^, conventional *R*-factors *R* are based on *F*, with *F* set to zero for negative *F*^2^. The threshold expression of *F*^2^ > σ(*F*^2^) is used only for calculating *R*-factors(gt) *etc*. and is not relevant to the choice of reflections for refinement. *R*-factors based on *F*^2^ are statistically about twice as large as those based on *F*, and *R*- factors based on ALL data will be even larger.

### Fractional atomic coordinates and isotropic or equivalent isotropic displacement parameters (Å^2^)

**Table:**

	*x*	*y*	*z*	*U*_iso_*/*U*_eq_	
N1	0.08963 (5)	0.5854 (3)	0.37586 (9)	0.0445 (4)	
O1	0.07135 (4)	0.7516 (3)	0.44261 (7)	0.0505 (3)	
O2	0.09612 (4)	0.3675 (3)	0.20520 (8)	0.0595 (4)	
H2	0.0848	0.4741	0.2407	0.089*	
O3	0.14491 (5)	0.0061 (3)	0.11078 (8)	0.0719 (5)	
H3	0.1213	0.1137	0.0890	0.108*	
C1	0.02835 (6)	0.9102 (4)	0.39001 (12)	0.0484 (4)	
H1A	0.0020	0.7819	0.3565	0.058*	
H1B	0.0375	1.0312	0.3411	0.058*	
C2	0.01029 (6)	1.0875 (4)	0.46377 (12)	0.0486 (5)	
H2A	−0.0158	1.2150	0.4287	0.058*	
H2B	0.0379	1.2027	0.5002	0.058*	
C3	0.12781 (6)	0.4381 (4)	0.41858 (12)	0.0444 (4)	
H3A	0.1401	0.4526	0.4865	0.053*	
C4	0.15231 (5)	0.2489 (3)	0.36349 (11)	0.0396 (4)	
C5	0.13584 (6)	0.2207 (4)	0.26086 (11)	0.0420 (4)	
C6	0.16024 (6)	0.0352 (4)	0.21112 (12)	0.0474 (4)	
C7	0.20050 (7)	−0.1205 (4)	0.26152 (13)	0.0531 (5)	
H7	0.2165	−0.2451	0.2277	0.064*	
C8	0.21755 (6)	−0.0930 (4)	0.36307 (13)	0.0534 (5)	
H8	0.2451	−0.1977	0.3971	0.064*	
C9	0.19369 (6)	0.0890 (4)	0.41324 (12)	0.0475 (4)	
H9	0.2052	0.1062	0.4812	0.057*	

### Atomic displacement parameters (Å^2^)

**Table:**

	*U*^11^	*U*^22^	*U*^33^	*U*^12^	*U*^13^	*U*^23^
N1	0.0463 (8)	0.0494 (9)	0.0400 (7)	−0.0006 (7)	0.0146 (6)	−0.0072 (7)
O1	0.0512 (7)	0.0609 (8)	0.0389 (6)	0.0100 (6)	0.0105 (5)	−0.0093 (6)
O2	0.0598 (8)	0.0715 (9)	0.0411 (6)	0.0174 (6)	0.0011 (6)	−0.0067 (6)
O3	0.0830 (9)	0.0906 (11)	0.0404 (7)	0.0200 (8)	0.0123 (6)	−0.0101 (7)
C1	0.0467 (9)	0.0537 (11)	0.0438 (9)	0.0022 (8)	0.0094 (7)	0.0009 (8)
C2	0.0488 (10)	0.0465 (11)	0.0524 (10)	0.0047 (8)	0.0158 (8)	0.0026 (8)
C3	0.0451 (9)	0.0513 (11)	0.0359 (8)	−0.0040 (8)	0.0088 (7)	−0.0031 (8)
C4	0.0384 (8)	0.0428 (10)	0.0379 (8)	−0.0055 (7)	0.0102 (7)	−0.0011 (7)
C5	0.0396 (9)	0.0452 (10)	0.0400 (9)	−0.0013 (7)	0.0077 (7)	0.0014 (8)
C6	0.0525 (10)	0.0513 (11)	0.0405 (9)	−0.0021 (9)	0.0153 (8)	−0.0034 (8)
C7	0.0529 (10)	0.0528 (11)	0.0586 (11)	0.0045 (9)	0.0232 (9)	−0.0016 (9)
C8	0.0454 (10)	0.0570 (12)	0.0578 (11)	0.0078 (9)	0.0124 (8)	0.0099 (9)
C9	0.0448 (9)	0.0565 (11)	0.0402 (9)	−0.0010 (8)	0.0083 (7)	0.0053 (8)

### Geometric parameters (Å, °)

**Table:**

N1—C3	1.277 (2)	C2—H2B	0.9700
N1—O1	1.4041 (16)	C3—C4	1.449 (2)
O1—C1	1.4377 (19)	C3—H3A	0.9300
O2—C5	1.3627 (19)	C4—C9	1.398 (2)
O2—H2	0.8200	C4—C5	1.403 (2)
O3—C6	1.3709 (19)	C5—C6	1.389 (2)
O3—H3	0.8200	C6—C7	1.369 (2)
C1—C2	1.505 (2)	C7—C8	1.389 (2)
C1—H1A	0.9700	C7—H7	0.9300
C1—H1B	0.9700	C8—C9	1.373 (2)
C2—C2^i^	1.521 (3)	C8—H8	0.9300
C2—H2A	0.9700	C9—H9	0.9300
			
C3—N1—O1	112.25 (12)	C9—C4—C5	118.48 (15)
N1—O1—C1	109.42 (11)	C9—C4—C3	119.58 (14)
C5—O2—H2	109.5	C5—C4—C3	121.94 (14)
C6—O3—H3	109.5	O2—C5—C6	116.75 (14)
O1—C1—C2	107.79 (13)	O2—C5—C4	123.26 (15)
O1—C1—H1A	110.1	C6—C5—C4	119.99 (15)
C2—C1—H1A	110.1	C7—C6—O3	118.65 (16)
O1—C1—H1B	110.1	C7—C6—C5	120.48 (15)
C2—C1—H1B	110.1	O3—C6—C5	120.87 (15)
H1A—C1—H1B	108.5	C6—C7—C8	120.18 (17)
C1—C2—C2^i^	113.41 (18)	C6—C7—H7	119.9
C1—C2—H2A	108.9	C8—C7—H7	119.9
C2^i^—C2—H2A	108.9	C9—C8—C7	119.96 (16)
C1—C2—H2B	108.9	C9—C8—H8	120.0
C2^i^—C2—H2B	108.9	C7—C8—H8	120.0
H2A—C2—H2B	107.7	C8—C9—C4	120.90 (16)
N1—C3—C4	121.39 (14)	C8—C9—H9	119.6
N1—C3—H3A	119.3	C4—C9—H9	119.6
C4—C3—H3A	119.3		
			
C3—N1—O1—C1	−179.64 (14)	O2—C5—C6—C7	−179.99 (15)
N1—O1—C1—C2	−179.36 (12)	C4—C5—C6—C7	−0.2 (3)
O1—C1—C2—C2^i^	−66.3 (2)	O2—C5—C6—O3	0.7 (2)
O1—N1—C3—C4	179.32 (13)	C4—C5—C6—O3	−179.56 (16)
N1—C3—C4—C9	−179.32 (15)	O3—C6—C7—C8	178.97 (17)
N1—C3—C4—C5	0.8 (2)	C5—C6—C7—C8	−0.4 (3)
C9—C4—C5—O2	−179.62 (14)	C6—C7—C8—C9	0.6 (3)
C3—C4—C5—O2	0.2 (3)	C7—C8—C9—C4	−0.2 (3)
C9—C4—C5—C6	0.6 (2)	C5—C4—C9—C8	−0.4 (2)
C3—C4—C5—C6	−179.53 (15)	C3—C4—C9—C8	179.71 (15)

Symmetry codes: (i) −*x*, −*y*+2, −*z*+1.

### Hydrogen-bond geometry (Å, °)

**Table:**

*D*—H···*A*	*D*—H	H···*A*	*D*···*A*	*D*—H···*A*
O2—H2···N1	0.82	1.94	2.648 (2)	145.
O3—H3···O2	0.82	2.26	2.706 (2)	115.
O3—H3···O1^ii^	0.82	2.26	2.930 (2)	139.

Symmetry codes: (ii) *x*, −*y*+1, *z*−1/2.
